# Heart Rate Detrended Fluctuation Indexes as Estimate of Obstructive Sleep Apnea Severity

**DOI:** 10.1097/MD.0000000000000516

**Published:** 2015-01-30

**Authors:** Eduardo Luiz Pereira da Silva, Rafael Pereira, Luciano Neves Reis, Valter Luis Pereira, Luciana Aparecida Campos, Niels Wessel, Ovidiu Constantin Baltatu

**Affiliations:** From the Center of Innovation, Technology, and Education—CITE (ELPS, VLP, LAC, OCB), Camilo Castelo Branco University (UNICASTELO), Sao Jose dos Campos Technology Park, Sao Jose dos Campos; University Iguaçu Campus—V (ELPS), Itaperuna, Rio de Janeiro; Department of Biological Sciences (RP), State University of Southwest Bahia—UESB, Jequie, Bahia; Sleep Institute of Itaperuna (LNR), Rio de Janeiro, Brazil; and Humboldt-Universität zu Berlin (NW), Berlin, Germany.

## Abstract

In the present study, we aimed at investigating a heart rate variability (HRV) biomarker that could be associated with the severity of the apnea–hypopnea index (AHI), which could be used for an early diagnosis of obstructive sleep apnea (OSA).

This was a cross-sectional observational study on 47 patients (age 36 ± 9.2 standard deviation) diagnosed with mild (23.4%), moderate (34%), or severe (42.6%) OSA. HRV was studied by linear measures of fast Fourier transform, nonlinear Poincaré analysis, and detrended fluctuation analysis (DFA)—DFA α1 characterizes short-term fluctuations, DFA α2 characterizes long-term fluctuations. Associations between polysomnography indexes (AHI, arousal index [AI], and oxygen desaturation index [ODI]) and HRV indexes were studied.

Patients with different grades of AHI had similar sympathovagal balance levels as indicated by the frequency-domain and Poincaré HRV indexes. The DFA α2 index was significantly positive correlated with AHI, AI, and ODI (Pearson *r*: 0.55, 0.59, and 0.59, respectively, with *P* < 0.0001). The ROC analysis revealed that DFA α2 index predicted moderate and severe OSA with a sensitivity/specificity/area under the curve of 0.86/0.64/0.8 (*P* = 0.005) and 0.6/0.89/0.76 (*P* = 0.003), respectively.

Our data indicate that the DFA α2 index may be used as a reliable index for the detection of OSA severity.

## INTRODUCTION

Obstructive sleep apnea (OSA) is the most common form of sleep apnea and it is characterized by partial or complete blockage of the upper airway during sleep. According to the frequency of apnea/hypopnea events per hour (ie, apnea–hypopnea index [AHI]) determined through polysomnography (PSG), OSA has been categorized to mild, moderate, or severe level of breathing and sleep disturbance.^1^ OSA is considered as a considerable risk factor for cardiovascular morbidity.^2,3^ Major pathophysiologic mechanisms responsible for cardiovascular complications associated to OSA include activation of the autonomic nervous system (ANS).^2^ The impact of OSA on ANS has been extensively studied and continues to be a subject of actual interest. The alterations of the cardiovascular ANS arise subsequently to the upper airway collapse-induced apneic and hyperventilation phases during sleep. Heart rate variability (HRV) algorithms have been used to study the cardiac ANS under various pathophysiological conditions for potential early diagnosis, prognosis, or risk stratification of diseases.^4,5^ These HRV algorithms were employed to investigate the ANS responses to different types of sleep apnea in normal and sleep apnea subjects^6,7^ and detect abnormal breathing events.^8–12^ Most of these studies analyzed the heart rate extracted from specific sleep segments (or time windows) associated with apnea/hypopnea events or different sleep stages. In the present study, we aimed at identifying a HRV biomarker that could be correlated with the severity of OSA, which could be directly detected from a full-length of sleep electrocardiogram (ECG) without the need of preliminary detection and segmentation in sleep apnea time windows. For this, besides linear algorithms, we employed nonlinear analyses of HR dynamics that are able to detect long-range correlations in noisy, nonstationary time series. Those included Poincaré plots and detrended fluctuation analysis (DFA) that may detect pathophysiologic information that is not achieved by conventional HR variability analysis.^13^

## MATERIALS AND METHODS

### Study Design and Setting

All subjects were referred to the Sleep Institute of Itaperuna, Rio de Janeiro, Brazil, for the investigation of possible sleep-disordered breathing. This was a cross-sectional observational study on 47 patients (age 36 ± 9.2 standard deviation [SD]) diagnosed through PSG with mild (23.4%), moderate (34%), or severe (42.6%) OSA between August 2012 and April 2013. Patient-level data were anonymized by removing all patient-identifying details and allocating a unique study code to each PSG recording. The study was approved by the Research Ethics Committee of the Faculty of Medical Sciences in accordance with resolution 196/96 and 340/2004 of the National Health Council (Ministry of Health) for research on human beings (CAAE: 01328712.0.0000.5244, permit no. 18326). The study is registered in ClinicalTrials.gov with the registration number: NCT02044900.

### Inclusion and Exclusion Criteria

Criteria of selection from the PSG database were males over 18 years of age diagnosed with OSA. Subjects with a history or clinical examination suggestive of hypertension, neuromuscular disease, or any surgical treatment were not considered in the study.

### Study Measurements: PSG

PSG monitoring and diagnosis was in accordance with the American Society of Sleep Medicine standards.^14^ Standard baseline PSG was performed using a computerized system (iBlue 42; iCelera – Celera Tecnologia em Equipamentos Médicos, Sao Paulo, Brazil). The following extracted signals were measured: 2 electroencephalogram channels (O1-A2, O2-A1, C3-A2, C4-A1), 2 electrooculogram channels, electromyography (EMG; bilateral anterior tibialis), pulse oximetry (arterial oxygen saturation), nasal–oral airflow (thermistors), and ECG derivation DII. The polysomnograms were scored according to the criteria of Rechtschaffen and Kales.^1^ Respiratory signals were scored automatically in accordance with current, internationally accepted guidelines.^15^ Obstructive apnea was defined as the complete cessation of or a >50% reduction in airflow lasting >10 seconds, accompanied by persistent respiratory effort, with or without oxygen desaturation or arousal. The AHI was defined as the number of obstructive hypopneas and apneas per hour of sleep. Hypopnea was defined as a reduction in airflow of <50% for >10 seconds, detected by thermistor or nasal cannula, accompanied by oxygen desaturation of ≥4% or an arousal of ≥1.5 seconds. Apnea was defined as an airflow reduction of ≥80% for at least 10 seconds. The oxygen desaturation index (ODI) was defined as the number of events per hour in which oxygen saturation decreased by ≥4%. Arousal was defined as any electroencephalography frequency shift lasting 3 to 15 seconds during non-rapid eye movement sleep; during rapid eye movement sleep, an increase in EMG was required as well. Arousal index (AI) was defined as the number of arousals per hour of sleep.

### Study Measurements: HRV Analysis

Single-lead DII ECG recordings were extracted from polysomnogram measurements. The duration of the recordings was 412 ± 12 minutes. HRV was assessed with the Kubios HRV analysis software (Department of Applied Physics, University of Eastern Finland).^16^ HRV was assessed in frequency domain by using a fast Fourier transform spectral analysis; the parameters included were as follows: low frequency (LF; 0.04–0.15 Hz), high frequency (HF; 0.15–0.4 Hz), and LF/HF ratio. We normalized LFnu and HFnu for analysis so as to minimize the effect on these 2 values by changes in the total power considering them as equivalent carriers of information about sympathovagal balance.^17^ The nonlinear properties of HRV were analyzed by Poincaré plots and DFA. In the Poincaré plots, an ellipse was fitted and 2 indexes were calculated: SD1 (the standard deviation of the points perpendicular to the line of identity) and SD2 (the standard deviation along the line of identity). In the DFA method, long-range correlations between interbeat intervals separated by several beats were detected by investigating the scaling behavior of the heartbeat fluctuations on different time scales disregarding trends and nonstationarities in the data.^18^ A detailed description of the DFA algorithm and its underlying theory for the analysis of neuronal oscillations was pertinently presented by Hardstone et al^13^ in their recent methods article. We quantified the fractal structure of heart rate by estimating a short-term (α1, short-term fluctuations, obtained from the range 4 ≤ n ≤ 16) and a long-term (α2, long-term fluctuations, obtained from the range 16 ≤ n ≤ 64) scaling exponent by DFA.

### Statistical Analysis

Data were tested for normality using the D’Agostino and Pearson omnibus normality test. Data normally distributed are represented by mean (standard error), and data that are not normally distributed are represented by median (interquartile range). Differences between 3 distinctive populations were tested using analysis of variance followed by Tukey's multiple comparisons test (*P* < 0.05 was regarded as being statistically significant). Pearson's correlation coefficient *r* was used to quantify a relationship between ≥2 variables; a 2-tailed *P* value <0.05 was considered statistically significant. A receiver-operator characteristic (ROC) analysis for the HRV index that correlated best with the AHI was performed. To evaluate the efficacy of the HRV index that correlated best with the AHI as a potential tool for diagnosing different grades of obstructive apnea, 2 ROC curves based on a threshold of AHI = 15 (for moderate obstructive apnea) and AHI = 30 (for severe obstructive apnea) were plotted and their areas under the curve were calculated as a measure of the overall efficacy of the diagnostic score. Data were analyzed using statistical software (Prism 6, GraphPad Software, Inc., La Jolla, USA for Mac OS X).

## RESULTS

### Significant Positive Correlation Between AHI and the Other PSG Indexes

**FIGURE 1:**
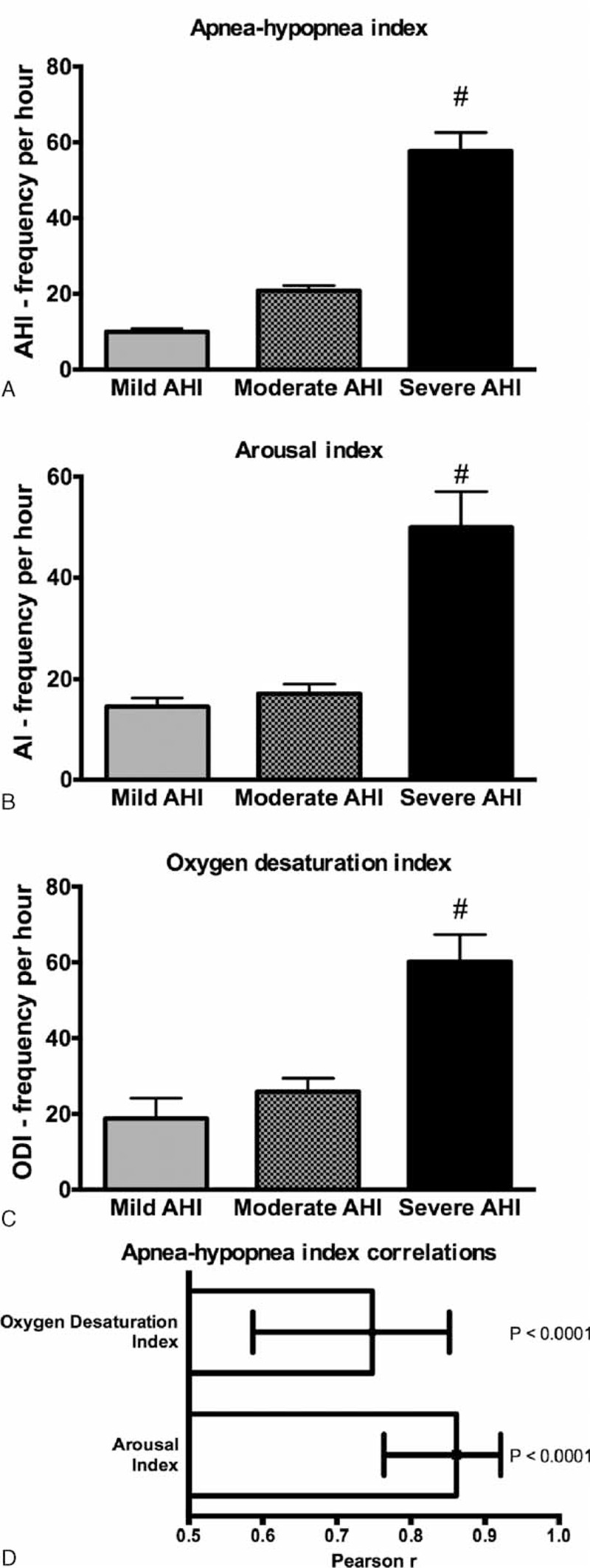
(A) AHI, (B) AI, and (C) ODI in obstructive sleep apnea across different degrees of severity according to the AHI: mild (AHI < 15), moderate (15 < AHI < 30), and severe (AHI > 30); #, *P* < 0.001. (D) Correlations between AHI and the other polysomnography indexes, AI, and ODI. Values are Pearson *r* and 95% CI. AHI = apnea–hypopnea index, AI = arousal index, CI = confidence interval, ODI = oxygen desaturation index.

The mild, moderate, or severe OSA was defined according to the AHI (Figure 1A–C). Multiple comparisons revealed significant differences between the severe group and the other 2 groups (mild and moderate) for all PSG indexes AHI, AI, and ODI. The AHI was significantly correlated with AI and ODI (Figure 1D, the Pearson correlation coefficients had values of 0.86 (95% confidence interval [CI]: 0.77 to 0.92) for the AHI to AI correlation and 0.75 (95% CI: 0.60 to 0.85) for the AHI to ODI correlation).

### Patients With Different Severity Levels of AHI Have Similar Basal Sympathovagal Balance Levels

**FIGURE 2:**
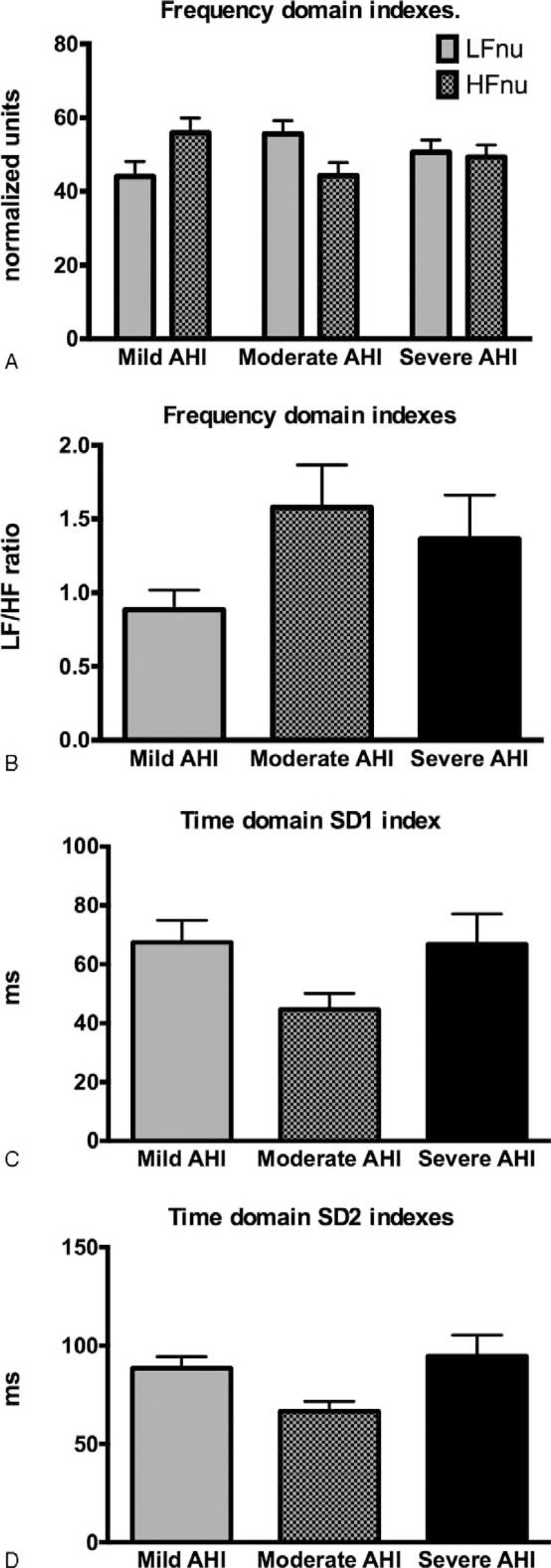
Basal sympathovagal balance levels indicated by (A) and (B) the frequency-domain HRV indexes, and the (C) and (D) time-domain HRV indexes in obstructive sleep apnea across different degrees of severity according to the AHI: mild (AHI < 15), moderate (15 < AHI < 30), and severe (AHI > 30). AHI = apnea–hypopnea index, HF = high frequency, HRV = heart rate variability, LF = low frequency, SD = standard deviation.

Patients with different grades of AHI had similar sympathovagal balance levels, as indicated by the HRV frequency and time-domain indexes. The LFnu, Hfnu, and LF/HF values among the different severity apnea groups were statistically not different (Figure 2A and B). The time-domain indexes SD1 (representing the dispersion of points perpendicular to the line of identity) and SD2 (representing the dispersion of points along the line of identity) were also not statistically different among the different severity apnea groups (Figure 2C and D).

### Heart Rate DFA Indexes Correlates in OSA

**FIGURE 3:**
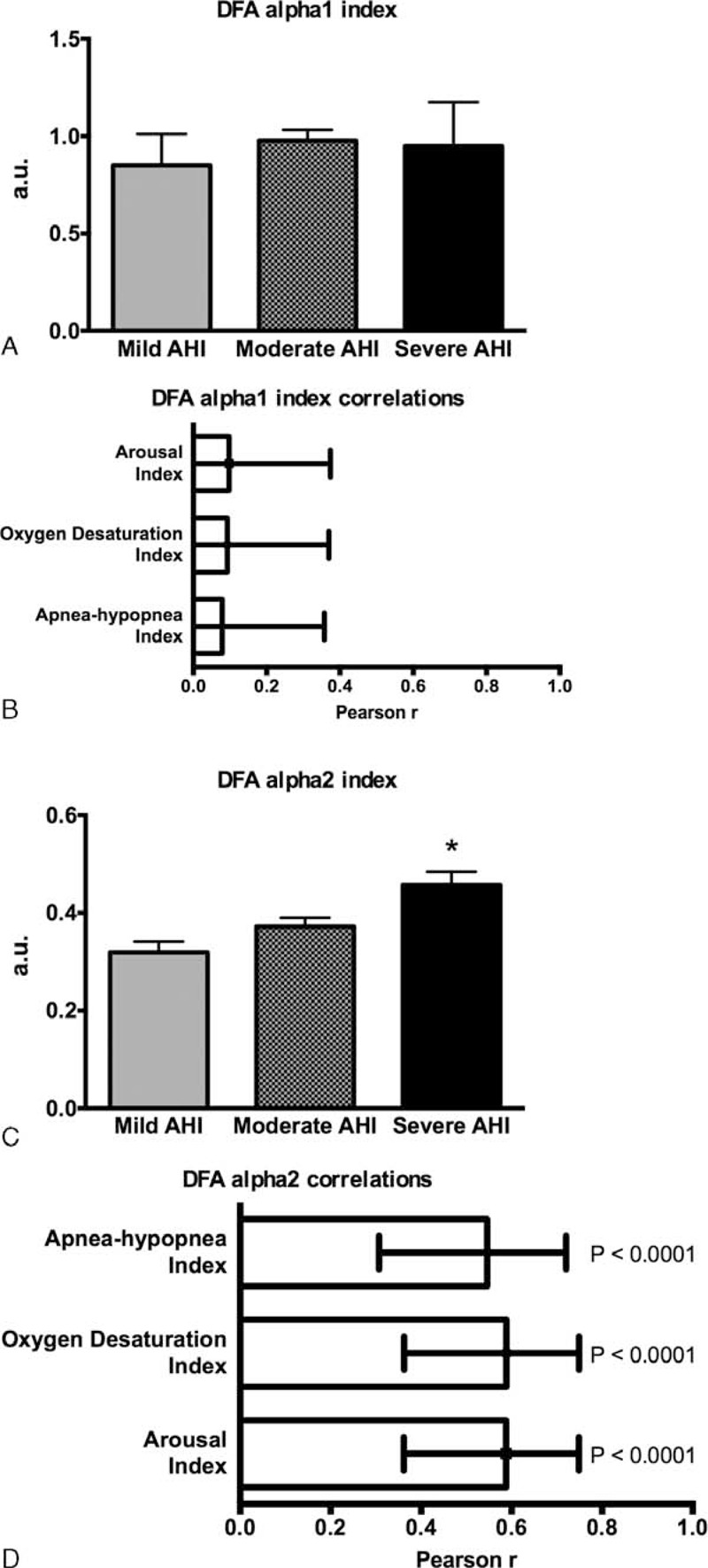
HRV DFA indexes in different degrees of obstructive sleep apnea and their correlations with polysomnography indexes. (A) and (B) DFA α1 index has no correlation with the AHI and the other polysomnography indexes. (C) and (D) DFA α2 index is significant positive correlated with the AHI and the other polysomnography indexes. AHI = apnea–hypopnea index, a.u. = arbitrary units, DFA = detrended fluctuation analysis, HRV = heart rate variability.

DFA α1 values were close to 1.0 (Figure 3A) indicating persistent long-range power-law correlations and suggesting that the fluctuations are generated by complex systems with multiple feedback regulations.^16,19^ The DFA α1 index was not significantly altered across different OSA severity categories (Figure 3A) and did not correlate with the AHI or with other PSG indexes (Figure 3B). DFA α2 values were close to 0.5 (Figure 3C) suggesting random dynamics (no correlation) of the R–R interval time series. There was a significant positive correlation of the DFA α2 index with the AHI and the other PSG indexes (Figure 3D).

### Heart Rate DFA α2 Index Prediction Value for the Severity of OSA

**FIGURE 4:**
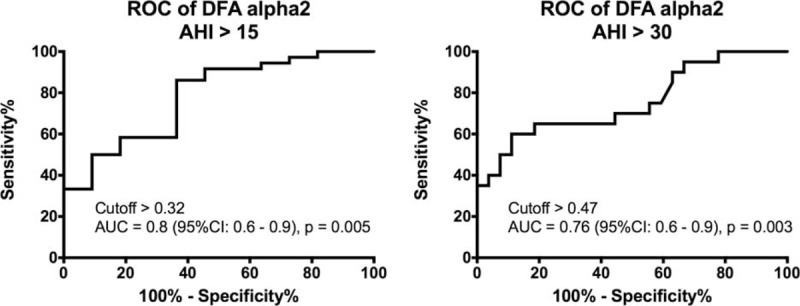
Diagnostic yield for DFA α2 index cutoff values compared with AHI cutoff values (reference values) obtained by polysomnography. ROC curve for identifying patients with (A) moderate (AHI threshold = 15) or (B) severe obstructive sleep apnea (AHI threshold = 30). AHI = apnea–hypopnea index, AUC = area under the curve, CI = confidence interval, DFA = detrended fluctuation analysis, ROC = receiver–operator characteristic.

ROC curves were examined to identify the optimal DFA α2 index thresholds that maximize sensitivity and specificity for predicting moderate or severe OSA. Thus, for moderate OSA, the threshold was set to AHI > 15 as dependent variable (Figure 4), using a cutoff for DFA α2 index > 0.32; sensitivity for moderate OSA prediction was 86.11% (95% CI: 70.50 to 95.33) and specificity was 63.64% (95% CI: 30.79 to 89.07). The calculated area under a ROC curve was 0.8 (95% CI: 63 to 94) with significance value of *P* = 0.005. Setting the threshold to AHI > 30 as dependent variable for severe OSA (Figure 4), the cutoff value for identifying severe OSA patients with DFA α2 index > 0.47 and yielded a sensitivity prediction of 60% (95% CI: 36.05 to 80.88) and specificity 88.89% (95% CI: 70.84 to 97.65). The calculated area under a ROC curve was 0.76 (95% CI: 60 to 90) with significance value of *P* = 0.003.

## DISCUSSION

The major finding of this study is that the heart rate-derived DFA α2 index from the full length of sleep ECG can accurately predict moderate or severe OSA. Also, there are significant positive correlations between AHI and the other PSG indexes, ODI and AI.

Agreements between AHI, ODI, and AI were found in our as well as in other studies.^20^ OSA is defined by episodes of partial or complete collapse of upper airway during sleep (ie, AHI). This causes a decrease or complete cessation of airflow. Consequently, a defective alveolar ventilation results and arterial oxygen saturation may drop (ie, ODI). These events are commonly terminated by arousals (ie, AI). Therefore, it makes sense to detect a statistical correlation between these PSG indexes that may be useful in standardizing PSG equipment and specific scoring approaches for quantifying sleep-related breathing disorders.^21,22^

In the present study, the frequency (LFnu, HFnu, and LF/HF ratio) and time-domain (SD1 and SD2) HRV indexes from the full length of sleep ECG were not different among the groups with different severity OSA. The normalized LFnu and HFnu frequency-domain indexes and their LF/HF ratio are considered to carry information about sympathovagal balance.^17^ The time-domain SD1 appears to be an index of instantaneous beat-to beat variability and SD2 index that characterizes long-term HRV is thought to reflect global autonomic variability.^4^ Although we did not find significant differences among the OSA groups in the frequency (LFnu, HFnu, and LF/HF ratio) and time-domain (SD1 and SD2) HRV indexes, these data do not disagree with several indications of activated sympathetic activity in specific narrow time windows when apnea/hypopnea episodes occur. In the present study, analyzing the HRV of a full-length sleep through linear analyses may give information on the baseline levels of the sympathovagal balance and may not have the power to detect short-term apnea/hypopnea events.

DFA measures the fractal-like correlation properties of the R–R interval time series.^23^ The long-term α2 exponent provides a relatively good approximation of the multiscale structure of beat-by-beat series of HR dynamics.^24^ As such, DFA α2 alterations, indicating loss of fractal HR dynamics, were significantly associated with the risk of cardiac death.^25^ Patients with OSA have increased risk of cardiovascular disease.^25^ In these patients, significant relationships between the risk of cardiac death and AHI were reported.^26^ In the present study, the significant positive correlation detected between DFA α2 and AHI may indicate that the alteration of fractal HR dynamics might represent the linkage of AHI on the prognosis of OSA. In a study with hemodialysis patients, DFA α2 was significantly positive correlated with depression score,^27^ which is a common complication in patients with OSA and a possible independent cardiovascular risk factor.

The ROC sensitivity analysis of α2 index indicated that the fraction of people with OSA at which the test correctly identifies as positive was 86.11% for moderate and 60% for severe OSA. The fraction of people without the disease at which the test correctly identifies as negative was 63.64% for moderate and 88.89% for severe OSA, as indicated by ROC specificity. Although the ROC analysis indicated a significant prognostic power of DFA α2 index for the severity of OSA, our study's drawback is not having a control group without OSA (ie, AHI < 5). The present study was limited by the data available in the patient sleep institute record. Further studies shall include volunteers without sleep apnea to further characterize the DFA α2 index as a prognostic biomarker for OSA and also for other types of apnea.

OSA is a common disease. Community-based studies using screening questionnaires tend to overestimate indications for PSG raising the legitimate question “When Does This Patient Have Obstructive Sleep Apnea?”^28^ Moreover, it is recommended that clinical examination for likelihood of OSA should be established prior to referring patients for definitive testing. Our study indicates that DFA α2 index derived from an overnight heart rate recording can be a good predictive biomarker of OSA. Prescreening of patients suspected with OSA with heart rate DFA α2 index may improve discrimination of sleep apnea. Advances in health self-monitoring technologies including heart rate monitoring through ECG or photoplethysmography methods offer a promising choice to provide a simplified technique for screening of OSA and possibly of sleep apnea, in general, in cardiologists’ daily practice.
